# Epidermal lamellar bodies, essential organelles for the skin barrier

**DOI:** 10.3389/fcell.2025.1597884

**Published:** 2025-07-08

**Authors:** Corinne Leprince, Michel Simon

**Affiliations:** Toulouse Institute for Infectious and Inflammatory Diseases (INFINITy), INSERM UMR1291, CNRS UMR5051, Toulouse University, Toulouse, France

**Keywords:** skin, epidermis, barrier, traffic, vesicle

## Abstract

Skin lamellar bodies are members of the Lysosome-Related-Organelle (LRO) family, characterized by specific features related to the skin’s primary function, i.e., protecting the body from external assaults while minimizing dehydration. In the uppermost living cell layers of the epidermis, the vesicles and tubulovesicular network that make up the « lamellar body system » as identified by electron microscopists, play a crucial role in maintaining the skin barrier. As a secretory compartment, lamellar bodies carry a variety of compounds that, when released in the extracellular space or exposed at the membrane, contribute to the unique hydrophobic structure of the upper epidermis (lipids and lipid metabolism enzymes), regulate desquamation (proteases and inhibitors) and provide anti-microbial defense. The molecular machinery involved in the biogenesis and trafficking of skin lamellar bodies is only beginning to be deciphered, including the Rab11A GTPase, the Myosin5B molecular motor, and the CHEVI complex. This later one is constituted of the Vps33B and VIPAR tethering molecules, whose mutations lead to the ARC and ARKID syndromes. Further studies are needed to identify the key molecules regulating the various stages of LB biogenesis, maturation and exocytosis. It is likely that some of these molecules will be shared with other members of the LRO family. These studies will further enhance our understanding of the relationships between lamellar body trafficking and skin barrier dysfunction.

## Introduction

The epidermis, the outermost protective layer of the skin, is a stratified epithelium primarily composed of keratinocytes and organized into four distinct cell layers. In the basal layer (stratum basale), located closest to the dermis, keratinocytes are proliferating cells responsible for tissue renewal. As they move towards the surface of the skin, these cells undergo a differentiation process and populate the upper layers namely, the stratum spinosum, stratum granulosum, and finally the stratum corneum. This uppermost layer which is the most essential for the skin’s barrier function, is made up of dead and rigid cells, called corneocytes, filled with keratin filaments and embedded in a hydrophobic lipid matrix. The composition of the stratum corneum is critically dependent on the underlying living cells, particularly the granular keratinocytes which behave like true secretory cells (Elias et al., 1998; Ishida-Yamamoto et al., 2018; Matsui and Amagai, 2015).

## Lamellar bodies, organelles defined by electron microscopy

**FIGURE 1:**
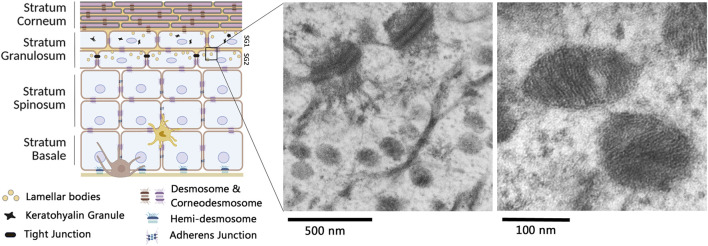
Lamellar bodies in multilayered epidermis. The epidermis is primarily constituted of keratinocytes, organized in various cell layers undergoing orderly differentiation. A melanocyte (brown cell) and a Langerhans cell (light brown cell) are also present in the epithelium. Lamellar bodies (yellow circles) are shown in keratinocytes of the Stratum Granulosum (SG) (left picture made with bioRender). By transmission electron microscopy, lamellar bodies can be observed at different scales: electron micrographs in the right part of the figure have been acquired by C. Leprince, from human skin biopsies as described in Reynier et al. (2019). Briefly, skin biopsies have been fixed with a mix of glutaraldehyde and paraformaldehyde, post-fixed with osmium tetroxyde, embedded in epoxy resin and post stained with uranyl acetate and lead citrate.

Epidermal lamellar bodies (LB), also known as lamellar granules, Odland bodies, or keratinosomes, were first identified by Odland in the 1960s as specific organelles of the skin (Odland, 1960). Their discovery was largely made possible by the development and availability of transmission electron microscopy. It allowed the visualization, in the uppermost living cell layers of the epidermis, of ovoid structures ranging from 50 to 200 nm in diameter, with a lamellar content and a surrounding membrane (Figure 1) (Landmann, 1988). Other organelles, also called lamellar bodies on the basis of their histological appearance, but much larger in size (around 1 µm in diameter), have been identified in alveolar type 2 epithelial cells. These later organelles have a specific function in the lung epithelium, preventing alveolar collapse upon breathing. Herein, in this paper focusing on skin biology, they will not be further analyzed.

Epidermal LB are first produced in the upper stratum spinosum and accumulate in the stratum granulosum (SG). Their biogenesis is closely linked to epidermal differentiation, and it is difficult to study them in 2D cultures of keratinocytes (Wertz, 2018). Electron microscopy images of the upper portion of the stratum granulosum often reveal deep invaginations of the plasma membrane containing a lamellar material, which presumably correspond to extrusion areas of the LB content into the extracellular space (Odland, 1960; Landmann, 1988; Odland and Holbrook, 1981). In 2001, L. Norlen suggested that LB are not discrete, ovoid vesicles, but instead form an extensive reticular network within the cytoplasm of the uppermost granular keratinocytes, creating a continuous membrane structure that extends from the cell interior to the extracellular space (Norlén, 2001). As a result, LB vesicles observed in traditional 2D electron micrographs might be viewed as planar projections of a tubuloreticular network. This alternative view has been partially confirmed over the following decades (Ishida-Yamamoto et al., 2018; Elias, 2005). In particular, the recent development of new electron microscopy methodologies combined with 3D reconstructions and the use of various sample preparation methods, which help minimize preparation-induced artefacts, has confirmed the presence of branched reticular strctures with a lamellar content in granular keratinocytes (den Hollander et al., 2016; Yamanishi et al., 2019). In a broader view of the stratified epidermis, Yamanishi et al. linked the LB morphology to keratinocyte differentiation. In 3D-reconstructed images of focused ion beam scanning electron micrographs (FIB-SEM), these organelles appear vesicular in the deeper granular layers (called SG3 as the third layer of stratum granulosum from the epidermal surface) and become reticular in the second layer of the stratum granulosum (SG2) (Yamanishi et al., 2019). In SG2 granular keratinocytes, it is interesting to note that the Trans-Golgi Network (TGN) is spread throughout the cytoplasm, with tubular structures connected to the similarly tubular LB.

Beyond the stuctural description, the debate on LB morphology has had implications for understanding the extrusion process into the extracellular space. A “membrane-folding” model proposed by L. Norlen views LB as a reticular structure that extends throughout a large part of the cytoplasm and is directly connected to the extracellular space, without the involvement of a barrier membrane (den Hollander et al., 2016; Norlén et al., 2022). The release of LB content is thought to occur through unfolding and folding of their membrane invaginations. An alternative “membrane trafficking” model, while incorporating the existence of a reticulo-tubular morphology, suggests that LB content is released into the extracellular space via a fusion process with the plasma membrane, similar to many other secretion pathways (Ishida-Yamamoto et al., 2018; Yamanishi et al., 2019; Elias, 2018). It is curently believed that the « LB system » consists of a combination of vesicles and reticular tubules, with the relative proportions of these components varying according to the state of granular cell differentiation and the need to maintain a functional barrier (Elias et al., 1998; Elias, 2005; Yamanishi et al., 2019; Madison, 2003; Menon et al., 2018). The reticular structure aligns with the plasticity and branched morphology of various subcellular compartments, such as recycling or late endosomes, and the TGN. Deciphering the releasing process will likely require more functional and dynamic approaches, such as models that induce barrier disruption. We may anticipate that future advancements in imaging technologies, including both electron or optical microscopy (the later enabling live imaging) will provide insights into this issue.

## The lamellar body cargoes

Once accumulated as a cytoplasmic pool, LB release their cargo into the extracellular space, on the apical side of granular keratinocytes (Yamanishi et al., 2019). The released compounds will contribute to the composition and structure of the stratum corneum, playing a crucial role in the skin’s barrier function. 3D-reconstructions of FIB-SEM electron micrographs have shown that LB begin secreting their contents in the second granular layer (SG2) with all granules being secreted in the first granular layer (SG1) (Yamanishi et al., 2019). The secretion process is most pronounced between SG2 and SG1, and sometimes biased towards one neighbor cell rather than another one, raising questions about the molecular and cellular factors controlling this release process (Ishida-Yamamoto et al., 2023).

**FIGURE 2:**
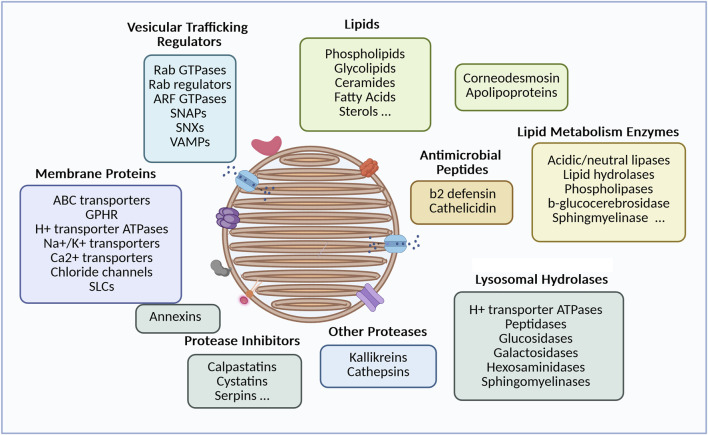
Diversity of lamellar body cargoes and related functions. The LB components detailed above come from data published by Wertz (2018), Wertz et al. (1984), Grayson et al. (1985), Vietri and Watt (2022) for the lipid part, and (Raymond et al., 2008) for the protein part. Components were grouped according to their biological function (picture made with bioRender).

Several groups have developped laborious methods to purify LB-enriched fractions, based on size or density criteria, and subsequently analyze their composition (Figure 2). Earlier reports revealed that they primarily consist of a mix of lipids, which aligns with the lamellar appearance of the organelle, and the essential role of lipids in the hydrophobic properties of the stratum corneum. Specifically, the contents include phospholipids, glycolipids (mostly glucosylceramides), free sterols and small amounts of sterol esters, ceramides and fatty acids (Wertz, 2018; Wertz et al., 1984; Grayson et al., 1985; Vietri and Watt, 2022). This unique lipid composition sets the skin apart from other tissues and likely serves a specialized function in the skin’s barrier. A particularly notable lipid species is made of unusual glucosylceramides with long ω-hydroxy-acyl fatty acid chains. These lipids are thought to be essential for lipid assembly within the organelles lumen and their anchoring to the surrounding membrane, which in turn influences the structure of lipid sheets in the inter-corneocyte spaces of the stratum corneum (Wertz, 2018; Wertz et al., 1984; Bowser et al., 1985). Once released into the extracellular space, the precursor lipids are metabolized by a series of hydrolytic enzymes, that have also been identified in LB-enriched fractions. These enzymes include glucosidases, galactosidases, phospholipase A, sphingomyelinase, hexosaminidase B, etc (Raymond et al., 2008). Based on a complex metabolic pathway, these enzymes generate non-polar lipids, such as ceramides, cholesterol, and free fatty acids (FFA), which are present in an approximately equimolar ratio in the stratum corneum and will organize into unique lamellar sheets (Elias, 2018; Schürer et al., 1991; Freinkel and Traczyk, 1985; Bouwstra et al., 2003; Bouwstra et al., 2023).

After LC-MS/MS analysis of a LB-enriched fraction, the proteomic study reported by Raymond et al. identified more than 900 proteins (Raymond et al., 2008). Although this enriched fraction may be contaminated by other subcellular constituents, it reveals several interesting elements beyond the lipid-processing enzymes, whose presence is also crucial for the skin barrier. First, the LB contains a series of membrane proteins, including ion channels and transporters. Among them is ABCA12, a member of the ABC surperfamily that facilitates the delivery of lipids into the organelle. Mutations in the ABCA12 gene have been identified as the cause of autosomal recessive congenital ichthyosis (ARCI), including Harlequin Ichthyosis, congenital ichthyosiform erythroderma, and lamellar ichthyosis, depending on the type of ABCA12 mutation. A defective ABCA12 results in a reduced lipid content in LB, as well as an abnormal LB morphology, a reduced pool of LB organelles, an impaired secretion into the extracellular space and a compromised barrier function that can be very severe in Harlequin Ichthyosis (Akiyama et al., 2003; Scott et al., 2013). In addition, LB-enriched fractions contain a structural protein called corneodesmosin which is essential for the rigidity of the stratum corneum (Simon et al., 1997). Corneodesmosin covers the external face of desmosomes, reinforcing the cohesion between corneocytes, the cell units of the statum corneum. Proteases from the kallikrein or cathepsin family as well as their inhibitors, such as LEKTI, cystatins or SLPI, are present in the LB lumen (Sondell et al., 1995; Ishida-Yamamoto et al., 2004). Once released into the extracellular space, these proteases play a role in desquamation, a process that involves the degradation of corneodesmosomes and is tightly regulated in the thickness of the stratum corneum. Several peptides (e.g., beta-2 defensin, cathelicidin) (Oren et al., 2003) or larger molecules like elafin are also part of the LB cargo, contributing to the innate antimicrobial protection. Lastly, chaperone molecules such as apolipoproteins, or caveolin (a scaffolding molecule for cholesterol and sphingolipids) have been documented in LB-enriched fractions (Raymond et al., 2008). It is likely that these molecules contribute to the molecular segregation within the organelles, although their precise roles and locations remain unclear. Molecular compartmentalization is evident in both classical electron micrographs and FIB-SEM images, where the organelle content can be heterogenous and not fully filled with a lamellar material (den Hollander et al., 2016; Raymond et al., 2008). This may prevent destructive, unfavourable molecular interactions, between lipids and their metabolizing enzymes, or between proteases and their inhibitors. Molecular segregation is also apparent in the heterogeneity of LB vesicles or sections of the tubular network. Immuno-electron microscopy has shown that different cargo markers occupy distinct domains within the LB structure, suggesting independent transport mechanisms (Ishida-Yamamoto et al., 2004). This was particularly observed for corneodesmosin and kallikrein 7, a distancing which might avoid premature proteolysis of corneodesmosin.

Finally, in view of their composition, skin LB are essential organelles for the homeostasis of the epidermal barrier: by creating an hydrophobic physical barrier (lipids and lipid metabolism enzymes), by reinforcing corneocyte cohesion (corneodesmosin), by controlling desquamation (proteases and inhibitors), and by providing an innate anti-microbial defence (antimicrobial peptides).

## Secretory vesicles, members of the lysosome related organelle (LRO) family

As shown above, the uppermost granular keratinocytes, particularly the second layer of granular keratinocytes (SG2), are crucial secretory cells for the skin barrier. In this context, LB are considered secretory vesicles that also share common features with members of the « Lysosome-Related-Organelle » (LRO, or ELRO for « endo-LRO ») family, which have been described in various cells and tissues (Marks et al., 2013; Delevoye et al., 2019). Like other LROs - such as lung lamellar bodies, melanosomes, Weibel-Palade bodies, lytic granules of cytotoxic lymphocytes, alpha granules of platelets - skin LB contain lysosomal acid hydrolases, including acid phosphatases, carboxypeptidases, cathepsins, and acid lipases. Additionally, LB have an acidic lumen, partly due to the presence of vacuolar-type proton pump ATPases, which are capable of transporting protons across the surrounding LB membrane (Raymond et al., 2008).

The various members of the LRO family have been shown to originate from different subcellular sites (Marks et al., 2013; Delevoye et al., 2019). Regarding skin LB, electron microscopy images typically describe budding sites originating from the TGN. Brefeldin A, a drug which causes the collapse of the Golgi complex, via inhibition of the exchange reaction on Arf-GDP, blocks LB formation (Feingold, 2007; Lippincott-Schwartz et al., 1989; Lippincott-Schwartz et al., 1991; Klausner et al., 1992). Additionally, the Golgi pH Regulator (GPHR) which acidifies the Golgi cisternae, has been suggested to contribute to the low pH of LB precursors that emerge from the TGN (Ishida-Yamamoto et al., 2018; Yamanishi et al., 2019; Madison, 2003; Tarutani et al., 2012; Sakai et al., 2007). In skin specific GPHR-knockout mice, Golgi function is disrupted, LB formation is impaired, and the epidermal barrier is compromised (Tarutani et al., 2012). Using immuno-electron microcopy, Ishida-Yamamoto et al. (2007) highlighted the presence of the Rab11 GTPase on tubulo-vesicular structures emerging from the TGN, which were stained with various lamellar body markers (corneosdesmosin, cathepsin D, LEKTI or Glucosyl-ceramides). Rab11 is a small GTPase found on recycling endosomes and the TGN, where it controls trafficking events between these intracellular compartments and the plasma membrane. Among the three Rab11 isoforms encoded by the mammalian genome - Rab11A, B and C–Rab11A was preferentially detected on corneodesmosin-stained LB, through high resolution (STED) confocal imaging (Reynier et al., 2016). Functional studies confirm the critical role of Rab11A GTPase as silencing RAB11A mRNA, in an in vitro 3D-model of reconstructed human epidermis, significantly impaired LB biogenesis (Reynier et al., 2016). As a result, stratum corneum lipids including the essential Cer-EOx ceramides with long carbon chains were reduced, the intercorneocyte lamellae were disorganized, and the epidermal barrier was compromised. These findings suggest that Rab11A controls LB biogenesis in skin granular keratinocytes, where it is particularly abundant and localized at the surface of LB. The identification of other key molecules in the LB trafficking pathway was extended to the well-known Rab11A effector, Myosin 5B, an actin-dependent molecular motor. Similar to Rab11A, Myo5B was detected on LB structures in granular keratinocytes. Silencing MYO5B mRNA decreased LB density, further inducing structural defects in the SC and impairing barrier function (Reynier et al., 2019). It has also been shown that the dynamics of the actin cytoskeleton play a crucial role in the distribution of LB throughout the cytoplasm of granular keratinocytes. To date, the role of microtubules and microtubule-dependent motors in LB motility remains under investigation. Taken together, these results suggest that immediately after the TGN exit, the funtional association between Rab11A, Myo5B and actin controls LB motility and potentially LB maturation through interaction and exchanges with endosomal compartments, such as recycling endosomes or late endosomes/lysosomes. This pathway closely resembles the transport mechanism of uroplakin vesicles in umbrella cells, another member of the LRO family (Khandelwal et al., 2009; Khandelwal et al., 2013).

**FIGURE 3:**
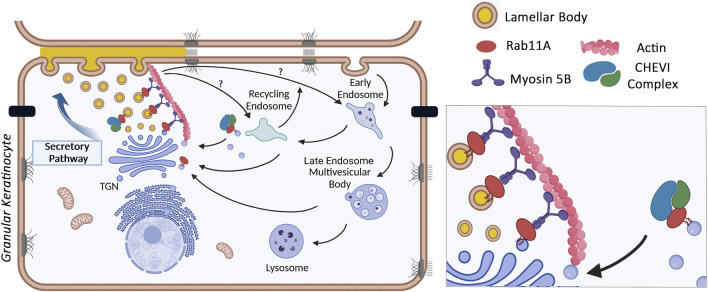
Control of LB trafficking in granular keratinocytes by the Rab11A GTPase, the Myosin 5B molecular motor and the CHEVI tethering compex (picture made with bioRender).

Further insights into LB trafficking have been gained through human genetics. ARC syndrome (Arthrogryposis, Renal dysfunction and Cholestasis; OMIM#208085) is an autosomal recessive multisystem disorder, which includes severe ichthyotic symptoms, caused by germline mutations in the VPS33B or VIPAR genes (Gissen et al., 2004; Hershkovitz et al., 2008). Subsequently, another mutation in the VPS33B was shown to cause the ARKID (Autosomal Recessive Keratoderma Ichthyosis Deafness; OMIM#620009) syndrome (Cullinane et al., 2010; Banushi et al., 2016; Gruber et al., 2017). VPS33B encodes a mammalian homolog of the yeast Vps33p (« vacuolar protein sorting 33 »). Together with the VIPAR protein (« VPS33B interacting protein involved in polarity and apical protein restriction »), VPS33B forms the CHEVI complex (« Complex C Homologues in Endosome-Vesicle Interaction »), which is involved in membrane tethering to a target compartment before interacting with a SNARE fusion complex. Interestingly, VPS33B and VIPAR are well known effectors of GTP-bound Rab11A. In the ARKID syndrome, the VPS33B mutation disrupts the interaction between VSP33B-VIPAR and Rab11A but does not affect the intrinsic interaction between the two components in the complex. In Hershkovitz et al. (2008), ultrastructural analysis of skin from ARC patients reported the presence of abnormal structures in corneocytes of the SC, which were suspected to be « entombed LB », likely due to defective LB biogenesis and/or secretion. This hypothesis was re-examined in tamoxifen-inducible VPS33B and VIPAR KO mice which replicate the ichthyotic phenotype of ARC, i.e., an abnormal structure of the SC and a defective epidermal barrier. In these mice, the expression of KLK5, one of LB compounds, was reduced in granular keratinocytes, when LB had an abnormal morphology, as observed by transmission electron microscopy. Additionally, neutral lipids were significantly reduced in the stratum corneum (Rogerson and Gissen, 2018). These studies suggest that the CHEVI complex plays a role in skin LB biogenesis and trafficking, similarly to its involvement in the biogenesis of another LRO, e.g., platelet alpha-granules. In haematopoietic cell-specific knockout mice, VPS33B deficiency results in a defect in alpha-granule biogenesis (Dai et al., 2016). In the epidermis, it remains to be determined whether the CHEVI complex plays its role on the surface of LB or on recycling endosomes, most likely through its membrane tethering function, which could influence LB maturation. Alternatively, it may regulate LB distribution by controlling apical polarity, as seen in hepatocytes (Figure 3), Rogerson and Gissen (2018), Bem et al. (2015), Rogerson and Gissen (2016).

## Additional genetic tools to decipher LB trafficking

Other loss-of-function mutations associated with an ichthyotic phenotype are still being investigated at the intersection of molecular genetics and skin biology. The defective molecules encoded by the mutated genes are suspected to be functionally linked to LB trafficking, although the molecular mechanisms remain unresolved.

For example, mutations in the SNAP29 gene cause CEDNIK syndrome (« CErebral Dysgenesis, Neuropathy, Ichthyosis, and Keratoderma», OMIM#609528), (Sprecher et al., 2005; Fuchs-Telem et al., 2011). The SNAP29 protein is a t-SNARE molecule, ubiquitously expressed and highly abundant in the epidermis, involved in membrane fusion processes. In the epidermis of CEDNIK patients, as well as in keratinocyte-specific SNAP29 knockout mice that replicate the ichthyotic symptoms of the human disease (Schiller et al., 2016; Keser et al., 2019), the epidermal barrier is disturbed, and LB morphology and secretion are abnormal. More specifically, the amounts of LB cargoes such as KLK7 or corneodesmosin are reduced in living keratinocytes, neutral lipids are much less abundant in the SC and ultrastructural images show remnants of non-degraded organelles in corneocytes. Whether SNAP29 affects LB trafficking, either directly or indirectly, warrants further investigation as SNAP29 has also been reported to control fusion processes between the autophagosome and endolysosome compartments (Diao et al., 2015; Tian et al., 2021). Interestingly, in a platelet-specific SNAP29 knockout mouse model, alpha-granule secretion is slightly decreased (Williams et al., 2016), which demonstrates in a cell type different from keratinocytes, that SNAP29 impacts the trafficking of another LRO. It is conceivable that alterations in the autophagosome or lysosome compartments, could impact the LB pool. In KO mice models, deletion of the autophagy-related atg7 gene induces secretion defects of either Weibel-Palade bodies in endothelial cells or lung LB in alveolar epithelium (Li et al., 2022; Torisu et al., 2013). In addition, other key players of autophagy, i.e., LC3B and ATG4B, have been shown to regulate melanosome motility and facilitate their intracellular positioning in melanocytes (Ramkumar et al., 2017). Presently, no functional link between epidermal LB and the autophagosome compartment have been supported by experimental data. But autophagosomes or autophago-lysosomes might contribute to LB maturation through exchange of membrane and luminal compounds, or to LB clearance in case of damaged LB.

MEDNIK syndrome (« Mental retardation, Enteropathy, Deafness, Neuropathy, Ichthyosis, Keratodermia” or IDEDNIK; OMIM#609313) is another multisystem genetic disorder caused by mutations in the AP1S1 gene, including an ichthyotic phenotype. AP1S1 encodes the σ subunit of the adaptor protein complex AP1, which is involved in clathrin coat assembly at TGN exit sites (Montpetit et al., 2008; Martinelli and Dionisi-Vici, 2014). Knockdown of the AP1S1 gene in a zebrafish model results in skin and neurologic defects, thereby reproducing major symptoms of the human disease and confirming the pathogenic role of the mutation (Montpetit et al., 2008). In certain tissues, such as brain and liver, AP1S1 deficiency has been shown to disrupt the intracellular distribution and trafficking of the ATP7A copper pump, affecting copper-dependent enzymes and copper homeostasis. However, no data is currently available on the impact of AP1S1 deficiency in the epidermis, on the trafficking of a given molecule which could impact the skin barrier. The use of the knockout zebrafish model would certainly be helpful in addressing this question.

The TMEM79 gene, also known as Mattrin, was originally identified in the Flaky tail (ft/ft) mouse line (Presland et al., 2000) a well-established model for atopic dermatitis. This mouse line carries two mutations, one in the Filaggrin (*Flg*) gene and another in the Tmem79/Mattrin (*Matt*) gene, the latter of which is responsible for the atopic phenotype (Sasaki et al., 2013; Saunders et al., 2013). Despite extensive studies in various tissues and pathologies, the precise function of the TMEM79 transmembrane protein remains unclear. In the skin, TMEM79 is expressed in hair follicles and in granular keratinocytes. Both Tmem79*^Ma/Ma^* mutant mice and Tmem79 *^−/−^* KO mice exhibit dermatitis-like skin inflammation, a defective skin barrier, and a desorganized SC. In Tmem79*^Ma/Ma^* mutants, Sazaki et al. (Sasaki et al., 2013) demonstrated that the levels of two LB cargoes, KLK5 and LEKTI, are reduced compared to wild-type mice, suggesting a role for TMEM79 in the control of LB trafficking (Elias, 2018; Sasaki et al., 2013). Additionally, another study reported a regulated interaction between TMEM79 and the cation channel TRPV3 (« Transient Receptor Potential Cation Channel 3 »), which is also expressed in keratinocytes (Lei et al., 2024). It was shown that TMEM79 could trap TRPV3 in the endoplasmic reticulum (ER) and facilitate its degradation in lysosomes, affecting TRPV3 function. These findings are similar to those described in HEK293T cells, where TMEM79 interacts with Frizzled at the ER membrane, controlling its ubiquitination by USP8 and consequently its degradation in the lysosomal compartment (Chen et al., 2020). In this report, Chen et al. suggest that TMEM79 is able to regulate the Wnt/Frizzled signalling, a biological pathway that might influence keratinocyte differentiation in the epidermis. Therefore, the interaction of TMEM79 with LB trafficking may involve the degradation or maintenance of key elements - controlling vesicular movements or intracellular signalling - although these elements still need to be identified.

## What the LRO family is teaching and what is still missing

The various molecular and cellular elements that regulate the structure of skin LB, their cargo and their fate in granular keratinocytes strongly support the idea that LB belong to the LRO family. These key regulatory elements are only beginning to be deciphered. Specifically, the identification of additional elements involved in LB maturation and exocytosis will be crucial, given their impact on the composition of the SC. As suggested by the proteomic analysis of LB-enriched fractions which includes more than 15 Rab GTPases, other Rab-family members and their effectors, not yet defined, may further control LB trafficking in the epidermis (Raymond et al., 2008). Some of them have been reported to regulate other LROs, such as Rab3D (van Weeren et al., 2004) or Rab38 (Osanai, 2018) on lung LB.

Menon et al. suggested that LB secretion occurs at low rates in a steady state epidermis, and is further stimulated under conditions of barrier disruption (Menon et al., 2018; Menon et al., 1994). After treating mouse skin with acetone which induces a barrier disruption, the authors observed an immediate release of LB cargoes and the disappearance of pre-formed LB from the cytoplasm of granular keratinocytes. This allowed a subsequent restoration of the skin barrier. The LB pool was reformed during a new phase of organellogenesis, which can last from 6 to 24 h (Elias et al., 1998; Menon et al., 1994). These experiments suggest that the process of LB secretion is tightly regulated in response to environmental variations, which aligns with the regulated trafficking of LRO, as documented in various cells and tissues. As with other LROs, a likely hypothesis is that LB secretion could be dependent on a cortical actin network and associated regulatory molecules, i.e., actin-binding proteins, membrane anchoring and fusion molecules. Rab family GTPases, including Rab27 or Rab3, which are known to be involved in exocytotic events in other cells and tissues, may also play a role (Fukuda, 2008). If this hypothesis is valid, the LB secretion process would be supported by a « membrane fusion » model rather than a « membrane folding » model, described in the first part of the paper. Interestingly, a cortical actin network enriched with corneodesmosin-labelled structures, which could serve as a docking site before LB exocytosis, has been commonly observed in 3D confocal imaging (Reynier et al., 2019).

It has been hypothesized that Ca^2+^ ions or cAMP are essential second messengers for the extrusion of skin LB (Menon et al., 1994; Denda et al., 2003). Indeed, in endothelial cells, high levels of intracellular Ca^2+^ have been reported to stimulate the exocytosis of Weibel Palade bodies following cell injury or stimulation with physiological agonists (Hordijk et al., 2024). In the stratified epidermis, the role of Ca^2+^ is both essential and complex, as calcium ions regulate many skin homeostatic pathways, including epidermal differentiation. The entire epidermis exhibits a Ca^2+^ gradient, with low levels in the basal and spinous layers, increasing Ca^2+^ levels in the granular layer and then declining again in the SC. In the stratum granulosum, the high Ca^2+^ concentration is primarily due to ion release from endoplasmic reticulum stores and, to a lesser extent, to Ca^2+^ influx from extracellular sources (Celli et al., 2010). These Ca^2+^ fluxes are controlled by various Ca^2+^ channels, pumps and Ca^2+^ sensor receptors, both at the membrane of the endoplasmic reticulum and at the plasma membrane (Lee et al., 1994; Lee et al., 1992; Lee and Lee, 2018). Presently, the dependence of LB exocytosis on Ca^2+^ signalling need to be examined in light of these new molecular insights, using biosensor technologies (Murata et al., 2018) and specific genetic knockouts of Ca^2+^-related molecules. Subsequently, it will be necessary to identify the calcium-sensitive proteins that translate the ion signal into LB trafficking and membrane fusion for exocytosis.

Additionaly, LB exocytosis is finely tuned in space. While electron micrographs reveal a broad distribution of LB in the cytoplasm of granular keratinocytes, their extrusion into the extracellular space seems to be confined to the apical membrane of these cells, which set up tight junctions in the SG2 layer (Ishida-Yamamoto et al., 2018; Yokouchi et al., 2016). This suggests that the local targeting of LB is tightly regulated, potentially by molecular complexes that are still undefined.

## Lamellar bodies in common skin diseases

Atopic dermatitis and psoriasis are two very common inflammatory skin diseases with distinct clinical manifestations, caused by a complex interplay between genetics, environmental factors, immune dysregulation, and skin barrier disruption (Tsai and Tsai, 2022). Both diseases show defects in epidermal differentiation, but relatively few studies have focused on the biogenesis and trafficking of lamellar bodies in the epidermis of patients.

Regarding psoriatic skin, Ghadially et al. (1996) provided evidence that a rare phenotype, i.e., erythroderma and active plaque psoriasis, display ultrastructural abnormalities at the epidermal level and compromised barrier function. Briefly, granular keratinocytes have an increased number of LB but most of them do not secrete their stored components. Consequently, the intercellular spaces of the stratum corneum are largely devoid of lamellae, and LB-like structures are retained in corneocytes. However, it should be noted that in the most common form of psoriasis, i.e., chronic plaque psoriasis, LB secretion and inter-corneocyte lipid structures are normal.

A greater number of studies have been conducted on atopic dermatitis. Both genetic and environmental factors play a role in the onset of the disease, which typically begins with a disruption of the skin barrier and an exacerbated inflammation. The compromised skin barrier is thought to facilitate the entry of allergens and/or antigens, activating both innate and adaptive immune responses, with a preponderant Th2-dependent inflammation. In turn, Th2 cytokines (e.g., IL-4 and IL-13) further impair the barrier by reducing the expression of key molecules involved in epidermal differentiation, such as proteins of cornification, lipid metabolizing enzymes, and antimicrobial peptides. Recent GWAS studies have identified atopic dermatitis susceptibility loci in genes encoding key molecules of the epidermal barrier (Budu-Aggrey et al., 2023). The most significant is the FLG gene, which encodes the structural protein filaggrin, essential for epidermal keratinization and hydration. While genetics alone cannot fully explain pathogenesis, it underscores the importance of molecules controlling the epidermal barrier. Focusing on LB and their cargoes, GWAS studies have highlighted the importance of genes encoding lipid metabolism enzymes, most of which are carried by LB. Additionally, atopic epidermis exhibit reduced levels of the three key SC lipids, ceramides, cholesterol and free fatty acids, including the ultra-long chain ceramides which are reduced in both lesional and non lesional atopic skin, leading to an abnormal lipid organization in the intercellular spaces of the SC (Bouwstra et al., 2003; Bouwstra et al., 2023; Ishikawa et al., 2010; van Smeden et al., 2016). This lipid defect, which contributes to a compromised skin barrier, has been associated with a reduced enzymatic activity as well as an altered LB secretion. Indeed, transmission electron micrographs of atopic epidermis have revealed, inside atopic corneocytes, abnormal LB-like structures which may be labelled with an anti-KLK7 antibody, indicative of an impaired extrusion process (Elias, 2018; Elias and Steinhoff, 2008; Igawa et al., 2017). To date, no key molecule involved in the LB trafficking process has been identified in genomic (GWAS), transcriptomic or proteomic studies. However, just as topical treatments based on ceramides are being explored for atopic dermatitis (Elias, 2018), ingredients that could stimulate LB secretion might improve, even partially, the barrier defects and help break the vicious circle of the chronic disease.

## Conclusion

Skin LBs are full and dedicated members of the LRO family. They share both morphological and functional characteristics with other family members. They also display unique features in accordance with the skin’s primary function: to protect the body from external aggressions while minimizing dehydration. Within the stratum granulosum, the vesicles and the tubulovesicular network that constitute the « LB system », as identified by electron microscopists, play a crucial role in the skin barrier function. As a secretory compartment, LB carry a variety of components adapted to the skin needs. Once released in the extracellular space or exposed at the membrane, the LB cargoes participate in the unique hydrophobic structure of the uppermost stratum corneum, as well as in the process of desquamation in this constantly renewing epithelium, and in providing an anti-microbial defense.

The molecular machinery involved in the biogenesis and fate of skin LB is only beginning to be deciphered. This is partly due to the stratified structure of the epidermis, where only the uppermost living layers contain LB structures, making experimental models more challenging. Some components of the molecular machinery, described so far, have been reported to regulate the trafficking of other LRO, in different cells or tissues. For example, Rab11A and Myosin 5B also control the trafficking of uroplakin vesicles in the urothelium, and the CHEVI complex also regulates the biogenesis of alpha granules in platelets. Because of this functional diversity, the dysfunction of any of these molecular components may affect various pathways in multiple tissues, potentially leading to syndromic diseases, as seen in ARC and ARKID syndromes.

Currently, further studies are needed to expand our understanding of skin LB. It is crucial to identify key molecules that regulate the different stages of LB biogenesis, maturation and exocytosis, as their dysfunction may lead to skin barrier defects, even in common skin diseases such as psoriasis and atopic dermatitis. In the era of « omic » data from the genome, transcriptome or proteome - also generated for the skin in various pathological conditions - it is essential to focus on the regulators and effectors of LB trafficking when the skin barrier is compromised. Human genetics has made a significant contribution to our understanding of the fate of LRO, including skin LB. Insights from a skin biology perspective could now contribute to improving the management of certain skin diseases.
